# *Porphyromonas gingivalis* Type 9 Secretion System Promotes Dysregulation of Vascular Smooth Muscle Cell Plasticity With Perturbed TGF‐β/Smad Signaling

**DOI:** 10.1002/mbo3.70044

**Published:** 2025-10-22

**Authors:** Priscilla L. Phillips, Leticia Reyes

**Affiliations:** ^1^ Microbiology & Immunology, Kirksville College of Osteopathic Medicine A. T. Still University of Health Sciences Kirksville Missouri USA; ^2^ Department of Pathobiological Sciences, School of Veterinary Medicine University of Wisconsin‐Madison Madison Wisconsin USA

**Keywords:** *P. gingivalis*/aortic smooth muscle cell interactions, *Porphyromonas gingivalis*, TGF‐β/Smad signaling, type 9 secretion system

## Abstract

The *Porphyromonas gingivalis* type 9 secretion system (T9SS) is known for secreting and anchoring protein cargos to the outer surface of the bacterium, which are then selectively packaged into outer membrane vesicles. We previously identified a link between *P. gingivalis*‐mediated dysregulated aortic smooth muscle cell (AoSMC) plasticity with binding of T9SS outer protein, PorU and select T9SS cargos to AoSMC proteins. To assess the role of T9SS in dysregulated AoSMC plasticity, a *PorU*‐deficient mutant was constructed in *P. gingivalis* strain A7UF. AoSMC was inoculated with sterile vehicle, wild‐type A7UF, or A7UFΔPorU and evaluated for proliferation, migration, and changes in the TGF‐β/Smad2/3 signaling axis. Deletion of *PorU* disrupted T9SS function in *P. gingivalis* A7UF. Loss of T9SS function impaired *P. gingivalis* invasion and persistence in AoSMC as well as attenuated microbial‐induced effects on AoSMC plasticity. Specifically, direct T9SS/AoSMC interactions were necessary for *P. gingivalis*‐induced AoSMC proliferation. Clarified supernatant from A7UF impaired the migration of infected AoSMC. *P. gingivalis* T9SS function was perturbed AoSMC TGF‐β/Smad3 signaling. Specifically, A7UF‐inoculated cells had increased linker and carboxy‐terminal phosphorylation of Smad3 that was attenuated in AoSMC inoculated with A7UFΔPorU. In summary, PorU and/or T9SS cargo play a role in *P. gingivalis*‐induced dysregulation of AoSMC plasticity as well as TGF‐β/Smad signaling. Microbial manipulation of host cell signaling events is important for cell differentiation and tissue remodeling and would constitute a new virulence function for T9SS.

## Graphical abstract

An intact type 9 secretion system (T9SS) is required for *Porphyromonas gingivalis*‐mediated dysregulation of aortic smooth muscle cell migration and proliferation. During infection, T9SS also enhanced phosphorylation of multiple sites on transcription factor Smad3 that is implicated in pathologic cell responses to TGF‐β/Smad signaling.

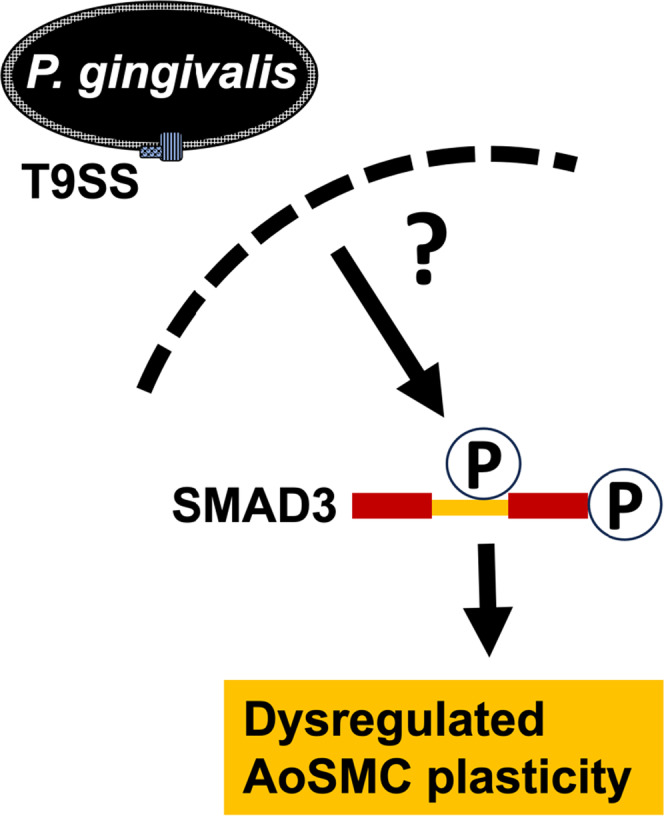

## 1 Introduction

*Porphyromonas gingivalis* is a Gram‐negative obligate anaerobe most known for its contribution to chronic periodontitis and a variety of systemic diseases (Z. Zhang et al. 2020), including vascular disorders, such as atherosclerosis Huang et al. (2023), aortic aneurysms (Aoyama et al. 2011; Nakano et al. 2011), and impaired uterine spiral artery remodeling during rat pregnancy (P. Phillips et al. 2018; Tavarna et al. 2022). Vascular smooth muscle cells, whether from arterioles or the large arteries, retain remarkable plasticity, and dysregulation of their plasticity underlies vascular pathogenesis (Lacolley et al. 2012; Petsophonsakul et al. 2019). Notably, dysregulated vascular smooth muscle cell phenotype switching is a feature of *P. gingivalis‐*associated vascular disorders (J. Zhang, Xie, et al. 2021; P. Phillips et al. 2018; Hokamura et al. 2010).

The TGF‐β/Smad signaling axis plays a central role in both physiologic and pathologic vascular smooth muscle cell plasticity (Rezaei et al. 2012; Low et al. 2019). Other signaling pathways such as notch‐, endoglin‐, and mitogen‐activated protein kinases (MAPKs) can modulate vascular smooth muscle cell responses to transforming growth factor β (TGF‐β) through crosstalk (Tian et al. 2017; Rezaei et al. 2012; Pedroza et al. 2020). Notably, *P. gingivalis*‐mediated effects on vascular smooth muscle cell plasticity are associated with TGF‐β, notch, and MAPK signaling (B. Zhang et al. 2013, 2016; L. Zhang et al. 2018; P. L. Phillips et al. 2022).

The type 9 secretion system (T9SS) was recently identified and found to be restricted to certain members of the *Bacteriodetes‐Chlorobi‐Fibrobacteres* superphylum (Lasica et al. 2017). The components and structure of the *P. gingivalis* T9SS are one of the best characterized (Veith et al. 2022; Song et al. 2022). One T9SS unit contains multiple secretion centers, and 0–4 T9SS per cell have been detected by in situ cryoelectron tomography (Song et al. 2022). A unique function of *P. gingivalis* T9SS is the processing, translocation, and anchoring of proteins (T9SS cargo) to the bacterial surface, whereby T9SS cargos can directly interact with host cells and/or extracellular matrix components, in vivo (Li et al. 2021). T9SS cargos are also packaged in outer membrane vesicles (OMVs) (Veith et al. 2022) that can diffuse into the local microenvironment or be transported to distant sites via the bloodstream (Z. Zhang et al. 2020). Specific *P. gingivalis* T9SS cargo proteins have been shown to assist with microbial adhesion/aggregation, nutrient acquisition, biofilm formation, and virulence, while others have unknown function (Veith et al. 2017). The impact of *P. gingivalis* T9SS has on the host is largely extrapolated from T9SS cargo with known function, such as gingipains and adhesins (Lasica et al. 2017). However, a recent study shows that the *P. gingivalis* T9SS modulates host inflammatory responses (Braun et al. 2022). Moreover, we previously reported a link between *P. gingivalis*‐mediated dysregulated aortic smooth muscle cell (AoSMC) plasticity with binding of select T9SS outer proteins and select T9SS cargos to AoSMC proteins (P. L. Phillips et al. 2022). Taken together, the interaction of T9SS outer surface components and/or T9SS cargos with AoSMC proteins may mediate host cell signaling events that perturb AoSMC phenotype switching.

The objective of this study was to determine if *P. gingivalis* T9SS disturbs AoSMC plasticity in vitro. To test our hypothesis, we inactivated T9SS function in *P. gingivalis* strain A7UF that we have shown disrupts rat vascular smooth muscle cell plasticity in vitro and in vivo (Tavarna et al. 2022; P. L. Phillips et al. 2022). *P. gingivalis* T9SS was inactivated by targeted deletion of outer surface protein PorU, which is essential for T9SS function (Braun et al. 2022; Glew et al. 2012; Mizgalska et al. 2021) and directly interacts with AoSMC proteins (P. L. Phillips et al. 2022). Herein, we show that the T9SS is important for *P. gingivalis*‐induced disturbance in AoSMC plasticity with perturbation of the TGF‐β/Smad signaling axis.

## 2 Methods and Materials

### 2.1 *P. gingivalis* Culture

All experiments were performed with the same working stock of *P. gingivalis* A7UF and A7UFΔPorU. A7UF is an oral isolate obtained from the Ann Progulske‐Fox laboratory (University of Florida) that we sequenced using an Ion‐Torrent platform (ION‐PGM) and de novo assembled using TorrentSuite software. Our assembled contigs showed approximately 95% and 96% alignment to *P. gingivalis* strains W83 and A7436, respectively (data not shown). Sequence alignments of our assembled contigs against *P. gingivalis* reference genome assemblies (downloaded from www.ncbi.nlm.nih.gov) were performed using Bowtie2. Fasta files of parent strain A7UF and A7UFΔPorU assembled contigs have been uploaded into the Zenodo repository (https://doi.org/10.5281/zenodo.14805777) and will be made available on request. For all experiments, all strains were cultured in a Coy Anaerobic chamber (Coylab.com) at the same time under the same conditions (37°C; 3%–5% H_2_, 3%–5% CO_2_, balance N_2_) in supplemented Tryptic Soy Broth (sTSB) [hemin (5 mg/L), yeast extract (5 g/L), l‐cysteine (0.5 g/L), and vitamin K1 or K3 (1 mg/L)] or supplemented blood agar (sBAP) [sTSB agar; 5% defibrinated sheep's blood]. A7UFΔPorU was grown on sBAP with 15 µg/mL erythromycin. Bacterial cultures underwent one passage on sBAP before passage to sTSB for in vitro inoculation of AoSMC.

### 2.2 Construction and Characterization of *P. gingivalis* Strain A7UFΔPorU

To create the PorU deletion mutant A7UFΔPorU, three sets of custom primers were designed to polymerase chain reaction (PCR) amplify regions upstream and downstream of the genomic sequence targeted for deletion (1425 bp) within *porU* and to the antibiotic selectable marker *ermF* encoded on the recombinant expression plasmid pUF4000 (Supporting Information File 1, Table S1). The primers were designed to allow PCR fusion of three PCR products, generating a final 2912 bp fusion amplicon where *ermF* (including its own promoter and terminator sequences) is encoded in reverse orientation with respect to the *porU* promoter (Supporting Information File 1, Figure S1).

After visual verification of the fusion PCR product on an agarose gel, the amplicons were purified and eluted with ddH_2_O using a filter column PCR clean‐up system and quantified by Nanodrop spectroscopy. Flash frozen electrocompetent *P. gingivalis* A7UF was prepared as described by Bélanger et al. (2007), and electroporated with ~0.5 µg of purified full‐length amplicons in a 2‐mm electroporation cuvette (Fisherbrand FB102), using a Bio‐Rad Gene Pulser set at 2.5 kV, 5.0 ms, 400 Ω, and 2.5 µF. Immediately after electroporation, 1 mL of freshly prepared and reduced culture media (sTSB) was added to the cells, and the suspension was incubated overnight anaerobically at 37°C to allow for chromosomal recombination and cell recovery. sBAP with erythromycin (15 µg/mL) or without (control) was spread with 150–200 µL of cell suspension and incubated anaerobically for 5–10 days at 37°C. As per our standard procedure, at least 24 positive clones resistant to erythromycin were streaked for isolation, cultured and freeze‐stored, and PCR assayed for *ermF* insertion and target deletion. For A7UFΔPorU, loss of pigmentation was indicative of *porU* deletion and verified by PCR. PCR‐verified positive clones were then selected for further verification by Sanger sequencing of gel‐purified long PCR amplicons containing the deletion mutation sequence and the flanking genes upstream and downstream of *porU*.

Using the classic strategy for mutant functional validation, we used a similar PCR fusion method as described above to create a complement clone to restore the *porU* gene in the mutant strain, using *TetQ* as the selectable marker to confer tetracycline resistance. However, we were unsuccessful after three attempts to restore *porU* in the mutant clone. It seemed that this failure was at least in part due to the A7UFΔPorU mutant's reduced ability to survive the electroporation process. Though slow to grow on solid media, especially from frozen cells, we confirmed that the prepared electrocompetent mutant cells were viable before electroporation and that there was a notable greater loss of viability after electroporation, compared with wild‐type A7UF. As an alternate strategy to validate the mutant, genomic DNA isolated from A7UFΔPorU was sent for whole genome sequencing to Novogene (Illumina Novaseq PE150). We used Trimmomatic and Spades software, which are publicly available in the Galaxy Toolbox (https://usegalaxy.org/), for de novo assembly of the Illumina PE150 reads into contigs. Artemis and Blast were used for targeted sequence analyses relevant to our study, including the region targeted for mutation, which verified that we made a clean mutation.

### 2.3 Preparation of *P. gingivalis* Inoculates

*P. gingivalis* from second passage sBAP cultures were grown to the stationary phase (18 h) in sTSB. Inoculates were prepared to achieve a relative multiplicity of infection (MOI) of 10 in sterile DMEM/F‐12 media containing 1% or 5% fetal calf serum. For all experiments, bacterial numbers in inoculates were confirmed by culture as previously described (P. L. Phillips et al. 2022). An MOI of 10 was chosen based on a previous study (B. Zhang et al. 2013) as well as a dose response study with A7UF and A7UFΔPorU (1000, 100, and 10 MOI), which showed AoSMC viability was not reduced after 24 h. Viability of AoSMC was evaluated by microscopy after staining with LIVE/DEAD Cell Imaging Kit (Catalog # R37601, Thermo Fisher Scientific) (Supporting Information File 1, Figure S1).

### 2.4 Preparation of *P. gingivalis* Clarified Supernatant

Clarified supernatant was prepared from stationary phase cultures (sTSB) as previously described (Furuta, Tsuda, et al. 2009). Briefly, both wild‐type A7UF and A7UFΔPorU were cultured at the same time, under the same conditions, in the same batch of sTSB, and were simultaneously harvested and processed. The bacterial number in each culture was determined by optical density readings and confirmed by serial dilution and plate culture as described (P. L. Phillips et al. 2022). The cultures were clarified by centrifugation at 10,000*g* for 30 min, then passed through a sterile polyethersulfone (PES) membrane 0.22‐µm filter (Thermo Fisher Scientific). Filtered supernatants that were used for AoSMC experiments were immediately aliquoted and stored at −80°C until use. Filtered supernatants from the same preparation that were saved for proteome studies received Halt protease and phosphatase inhibitors cocktail with ethylenediaminetetraacetic acid (EDTA) (Catalog # 78440, Thermo Fisher Scientific) before storage at −80°C.

The optimal amount of supernatant to be used for subsequent AoSMC experiments was determined by a dose response study in which AoSMC was incubated for 24 h with varying concentrations of clarified supernatant. A volume corresponding to an MOI of 40 (based on the original cultures equivalent to 4.7 μg/mL of total protein) was selected for final experiments since it was the highest MOI equivalent in which 99% of AoSMC remained viable after 24 h.

### 2.5 Proteomic Analysis of *P. gingivalis* Clarified Supernatants

To determine if the T9SS attachment complex proteins PorUVZQ (PG0026, PG0027, PG1604, and PG0602) were expressed, we evaluated the qualitative differences in the proteomes of filtered sTSB (control) and clarified supernatants from A7UF and A7UFΔPorU (*n* = 1) identified by nanoliquid chromatography coupled with tandem mass spectrometry/mass spectrometry (NanoLC‐MS/MS) as previously described (P. L. Phillips et al. 2022) with the following modifications. Samples were precipitated with cold acetone (80% final vol:vol) for 30 min at −20°C and pelleted by centrifugation at 16,000*g* for 10 min. Precipitated proteins were washed once with cold acetone, then once with cold methanol. Protein extracts were resolubilized and denatured in 50 μL of 8 M urea in 50 mM NH_4_HCO_3_ (pH 8.5). Protein concentrations were determined by using the Bicinchoninic Acid (BCA) assay, and 50 µg of each protein sample was used for tryptic/LysC digestion. For the reduction step, samples were first diluted to a final volume of 60 µL with 25 mM dithiothreitol (DTT) and 25 mM NH_4_HCO_3_ (pH 8.5) and incubated at 56°C for 15 min. After cooling on ice to room temperature, 3 μL of 55 mM chloroacetamide was added for alkylation, and samples were incubated in the dark for 15 min at room temperature. This reaction was quenched by the addition of 8 μL of 25 mM DTT. Subsequently, 15 μL of trypsin/LysC solution [100 ng/μL 1:1 trypsin (Promega): LysC (FujiFilm) mix in 25 mM NH_4_HCO_3_] along with 14 μL of 25 mM NH_4_HCO_3_ (pH 8.5) was added to the samples for a final 100 µL volume. Digests were carried out overnight at 37°C and then terminated by acidification with 2.5% trifluoroacetic acid to a final concentration of 0.3%.

To prepare samples for NanoLC‐MS/MS, digests were desalted using Pierce C18 SPE pipette tips (100 µL volume) per manufacturer protocol and eluted in 20 µL of 70/30/0.1% acetonitrile (ACN)/H_2_O/trifluoracetic acid (TFA). Samples were dried in a speed‐vac then reconstituted in 60 µL of 0.1% formic acid. Peptides were then analyzed by NanoLC‐MS/MS using the Agilent 1100 nanoflow system (Agilent) connected to a hybrid linear ion trap–orbitrap mass spectrometer (LTQ‐Orbitrap Elite, Thermo Fisher Scientific) equipped with an EASY‐Spray electrospray source (held at constant 35°C). Chromatography of peptides before mass spectral analysis was accomplished using a capillary emitter column (PepMap C18, 3 µM, 100 Å, 150 × 0.075 mm, Thermo Fisher Scientific) onto which 2 µL of extracted peptides was automatically loaded. NanoHPLC system delivered solvents: A, 0.1% (v/v) formic acid and B, 99.9% (v/v) acetonitrile, 0.1% (v/v) formic acid at 0.50 µL/min to load the peptides (over a 30 min period) and 0.3 µL/min to elute peptides directly into the nanoelectrospray with gradual gradient from 0% (v/v) B to 30% (v/v) B over 150 min and concluded with 10 min fast gradient from 30% (v/v) B to 50% (v/v) B at which time a 7‐min flash‐out from 50% to 95% (v/v) B took place. As peptides eluted from the HPLC column/electrospray source survey, MS scans were acquired in the Orbitrap with a resolution of 120,000, followed by CID‐type MS/MS fragmentation of the 30 most intense peptides detected in the MS1 scan from 350 to 1800 m/z; redundancy was limited by dynamic exclusion.

Elite acquired MS/MS data files were converted to mgf file format using MSConvert (ProteoWizard: Open‐Source Software for Rapid Proteomics Tools Development). Resulting mgf files were used to search against *P. gingivalis* proteome databases (UP00000588 04/05/2021 download, 1980 total entries) along with a cRAP (Common Repository of Adventitious Proteins) database of common contaminants (116 total entries) using in‐house Mascot search engine 2.7.0 [Matrix Science] with fixed cysteine carbamidomethylation and variable methionine oxidation plus asparagine or glutamine deamidation. Peptide mass tolerance was set at 10 ppm and fragment mass at 0.6 Da. Protein annotations, significance of identification, and spectral‐based quantification were done with Scaffold software (version 4.11.0, Proteome Software Inc., Portland, OR). Peptide identifications were accepted if they could be established at greater than 87.0% probability to achieve a False Discovery Rate (FDR) of less than 1.0% by the Scaffold Local FDR algorithm. Protein identifications were accepted if they could be established at greater than 20.0% probability to achieve an FDR less than 1.0% and contained at least two identified peptides. Protein probabilities were assigned by the Protein Prophet algorithm (Nesvizhskii et al. 2003). Proteins that contained similar peptides and could not be differentiated based on MS/MS analysis alone were grouped to satisfy the principles of parsimony. Proteins sharing significant peptide evidence were grouped into clusters.

### 2.6 AoSMC Cultivation

Primary AoSMC was isolated from adult‐specific pathogen‐free Sprague–Dawley rats (RRID: RGD_734476). Cells were collected under the approved protocol # V005576, University of Wisconsin Institutional Animal Care and Use Committee.

AoSMC was isolated as previously described (P. L. Phillips et al. 2022) using the double‐collagenase method (Villa‐Bellosta and Hamczyk 2015). Collagenase type 2 (Worthington Biochemical Corp., Catalog # LS004174) was used for tissue dissociation. For all experiments, AoSMC was initially prepared by culturing in DMEM/F‐12 (Catalog # 12‐719F, Lonza, Walkersville, MD, USA) with heat‐inactivated 10% fetal calf serum (Catalog # FB‐12, Omega Scientific) and gentamicin‐sulfate with amphotericin‐B (Catalog # CC‐4083, Lonza, Walkersville, MD, USA). For consistency, the same lot of fetal calf serum was used for all experiments. The purity of AoSMC was ≥ 90%, which was verified by positive staining for α‐smooth muscle actin (RRID: AB_2223019).

Passage 7 AoSMCs were used for all experiments. To synchronize the AoSMC division, cells were cultured under serum depletion (1% fetal bovine serum [FBS]) for 24 h in Dulbecco's modified Eagle's medium (DMEM)/F‐12 medium (without antibiotics). One percent FBS was needed to maintain the cell viability of our primary cells while maximizing the potential of capturing the cell responses to our pathogen with minimal serum interference. To study direct effects between bacteria and host cells, sterile medium or 10 MOI of *P. gingivalis* strains were prepared in DMEM/F‐12 containing either 1% or 5% FBS concentrations. Specifically, AoSMCs that were used for the TGF‐β Phospho Antibody Array were inoculated with media containing 1% FBS after serum starvation and maintained in 1% FBS until cell harvest. For all other experiments, AoSMCs received inoculates prepared in 5% FBS after serum starvation. The indirect effects of bacteria on AoSMCs were evaluated using two methods. The first was by Transwell assay, whereby bacterial cultures were separated from AoSMC by a Thincert 0.4‐µm pore size polyethylene terephthalate membrane (Catalog # 665641, Greiner bio‐one, www.gbo.com). The second method utilized clarified supernatants from A7UF or A7UFΔPorU at a volume corresponding to 40 MOI based on the original culture supernatant that was equivalent to 4.7 μg/mL of total protein per sample.

### 2.7 Assessment of *P. gingivalis* Colonization, Persistence, and Invasion in AoSMC

For all experiments, AoSMC was seeded at a cell density of 1 × 10^5^ cells per well in six‐well plates and allowed to adhere overnight to culture plates at 37°C and 5% CO_2_ (v/v). Adhered cells were then serum‐starved (1% FBS in DMEM/F‐12) for an additional 24 h before inoculation to synchronize the cell cycle. To further synchronize the timing of bacterial attachment to AoSMC, the cells were incubated on ice for 10 min before inoculation with 10 MOI of *P. gingivalis* prepared in DMEM/F‐12 with 5% FBS. The time that cells were placed in the incubator was considered time zero, and cells were incubated at 37°C and 5% CO_2_ (v/v) until harvest. For colonization experiments, AoSMC and corresponding culture fluid suspension supernatant were collected, harvested at 2.5‐, 6‐, and 24‐h postinoculation (PI), and cultured for *P. gingivalis*. For invasion and persistence assays, inoculated AoSMCs underwent a pulse antibiotic treatment at 1.5‐h PI designed to kill remaining extracellular bacteria as previously described (Reyes et al. 2013). Briefly, cell cultures were washed three times with sterile phosphate‐buffered saline (PBS) and pulse treated with 10 mg/mL ampicillin + 400 µg/mL metronidazole diluted in DMEM/F‐12 with 5% FBS for 1 h at 37°C and 5% CO_2_ (v/v). At 2.5‐h PI, AoSMC culture supernatants were cultured onto sBAP to confirm that bacteria were killed. Pulse‐treated cells (collected at 2.5 h) were then washed three times with antibiotic‐free PBS, followed by one wash with sterile water, then lysed with sterile water for 20 min at 37°C and 5% CO_2_ (v/v). Complete cell lysis was confirmed by microscopy. Wells containing pulse‐treated cells that were to be harvested at the 6‐ and 24‐h time‐point were washed three times with sterile PBS before the addition of antibiotic‐free DMEM/F‐15 with 5% FBS for continued incubation. For enumeration of live bacteria, all AoSMC supernatants and cell lysates collected at harvest were serially diluted 10‐fold in sterile PBS and cultured on sBAP.

### 2.8 Assessment of AoSMC Plasticity—Proliferation Assays

AoSMC plasticity was assessed by induction of proliferation as previously described (P. L. Phillips et al. 2022). AoSMC proliferation was measured by counting the total number of cells after 24 h of infection or by microscopy using detection of nuclear Ki67 as an indicator of cell proliferation. Briefly, an equal number of passage‐7 AoSMC was seeded in multiwell culture plates. After cells were allowed to attach to the plate overnight, they were serum‐starved (1% FBS in DMEM/F‐12) for 24 h to synchronize the cell cycle before direct inoculation with sterile media (DMEM/F‐12 with 5% FBS), or 10 MOI of A7UF or A7UFΔPorU and incubated for 24 h at 37°C and 5% CO_2_ (v/v). At the end of the incubation period, AoSMC was detached with 0.25% trypsin and counted with a hemocytometer. For detection of Ki67, AoSMC was seeded onto 12‐well plates (Greiner bio‐one wwwgbo.com) or chamber slides (NUNC Lab‐Tek II Chamber Slide system (Thermo Fisher Scientific, Catalog # 154534) and inoculated as described. At the end of the experiment, AoSMC was fixed with 4% buffered formalin for 10 min, washed with PBS, and permeabilized with 0.5% Triton‐X for 10 min. Cells were incubated for 30 min with blocking solution [2% goat serum, 1% bovine serum albumin, 0.1% Triton X‐100, 0.05% Tween 20, and 0.05% sodium azide in 0.01 M PBS] before addition of rabbit anti‐Ki67 antibody (Catalog # PA5‐19462, RRID: AB_10981523, Thermo Fisher Scientific) and mouse anti‐alpha actin (Catalog # ab76549, RRID: AB_2223019, Abcam). Isotype controls were stained with normal rabbit serum (Catalog # 31883, Thermo Fisher Scientific) and mouse Immunoglobulin G (IgG) isotype control (Catalog # 31903, Thermo Fisher Scientific). All antibodies were diluted 1:200 in blocking buffer. Goat antirabbit ALEXA 647 (Catalog # A‐21244, Life Technologies, Grand Island, NY) and goat antimouse ALEXA 594 (Catalog # A‐11032, Life Technologies, Grand Island, NY) were used for detection, and nuclei were counterstained with 4′,6‐diamidino‐2‐phenylindole (DAPI) (Catalog # P36934, ProLong Gold Antifade Mountant with DAPI, Thermo Fisher Scientific). For each biological replicate, 10 calibrated images that spanned the chamber area were captured with an EVOS Auto FL imaging system. Percent proliferating cells were calculated by dividing the number of cells in which the nucleus was Ki67 positive by the total number of DAPI‐positive nuclei per well.

To assess indirect effects of *P. gingivalis* on AoSMC, bacterial suspensions were added to upper chamber inserts with a 0.40‐µm permeable membrane (Catalog # 665641, Greiner bio‐one wwwgbo.com). Alternatively, AoSMC was inoculated with sterile medium (control) or A7UFΔPorU that was supplemented with clarified supernatant from A7UF or A7UFΔPorU (4.7 μg/mL of total protein per sample). As a positive control, AoSMC was directly inoculated with 10 MOI of A7UF. After 24 h, AoSMC was fixed and stained with the proliferation marker Ki67 and counted as described above.

### 2.9 Assessment of AoSMC Plasticity—Migration Assays

AoSMC plasticity was assessed by the induction of migration. AoSMC Migration assays were performed as previously described (Brown et al. 1994) with the following modifications. AoSMC was serum‐depleted for 24 h in 1% FBS in DMEM/F‐12 media to synchronize the cell cycle before inoculation with sterile medium, A7UF, or A7UFΔPorU suspended in 5% FBS in DMEM/F‐12 media. After an additional 24 h, adherent cells from all treatment groups were removed by 0.25% trypsin treatment, washed once with sterile PBS, counted, pelleted by centrifugation (800*g*), and resuspended in 5% FBS in DMEM/F‐12 to achieve a cell density of 1 × 105 cells/mL. For each treatment group, 10^4^ cells were inoculated into 8 mm Transwell inserts (Falcon Cell Culture Inserts by Corning, Catalog # 353097). To stimulate migration, bottom wells contained 3 µg/mL of Vitronectin (Catalog # 5051, Advanced Biomatrix.com) prepared in DMEM/F‐12 + 5% FBS. AoSMCs were incubated at 37°C and 5% CO_2_ (v/v) for 6 h before harvest. Cells within the top side of the Transwell chamber were removed before migrated cells (bottom side) were fixed with 4% buffered formalin. Fixed cells were washed with PBS and incubated for 30 min with blocking solution, then incubated overnight with mouse antismooth muscle actin (Abcam Cat # ab76549, RRID: AB_2223019) diluted 1:200 in blocking buffer. Goat antimouse ALEXA 594 (Catalog # A‐11032, Life Technologies, Grand Island, NY) was used for detection. Stained membranes were carefully removed from the Transwell and mounted to glass slides with ProLong Gold Antifade Mountant with DAPI. Calibrated images of the entire membrane were captured with an EVOS AutoFL imaging system, and total cell numbers were normalized by the surface area of the Transwell membrane (mm^2^).

### 2.10 Assessment of TGF‐β/Smad Signaling Axis in AoSMC

To determine what pathways involved in TGF‐β/Smad signaling are perturbed by *P. gingivalis*, whole AoSMC Lysates were evaluated with a TGF‐β Phospho Antibody Array (Full Moon Biosystems Inc., Sunnyvale, CA, USA, https://www.fullmoonbio.com). This array system simultaneously examined unphosphorylated and phosphorylated proteins involved in canonical and independent TGF‐β signaling pathways as well as downstream effectors of bone morphogenetic protein, Smad 1/5/8. The array was originally designed for human cells, but it recognizes 87% of rat proteins. To minimize the potential effects of growth factors in FBS, AoSMC cultures were maintained in 1% FBS in DMEM/F‐12 media throughout these assays.

AoSMC was serum‐depleted for 24 h in 1% FBS in DMEM/F‐12 media to synchronize the cell cycle before inoculation with sterile medium (control), *P. gingivalis* A7UF, or A7UFΔPorU suspended in DMEM/F‐12 medium with 1% FBS at an MOI of 10. At 24‐h PI, AoSMC supernatants were collected and checked for viable bacteria by culture as already described. AoSMCs were washed with ice‐cold PBS three times, then lysed with RIPA lysis and extraction buffer (Catalog # 89900, Thermo Fisher Scientific) containing Halt protease and phosphatase inhibitors cocktail with EDTA (Catalog # 78440, Thermo Fisher Scientific). Samples were stored at −80°C until processing for array analysis. Each TGF‐β array experiment consisted of pooled samples made up of two biological replicates from two independent experiments. Protein quality and concentration in pooled samples were determined by ultraviolet absorbance spectroscopy (A280). Biotin labeling of protein samples, blocking, and labeling of slide arrays were performed according to the manufacturer's instructions. Labeled slides were scanned and analyzed by Full Moon Biosystems. Analysis of each slide consisted of determining the signal intensity for each spot as well as the average signal intensity and coefficient of variation for replicate spots. To determine differences between treatments, ratios of each protein in each sample were generated by comparing the phospho‐specific antibody's signal to its site‐specific antibody's signal (i.e., phosphorylated amino acid signal/corresponding unphosphorylated amino acid signal). Results were reported as fold change (ratios) between control and treated samples (i.e., A7UF/uninfected control, A7UFΔPorU/uninfected control, and A7UFΔPorU/A7UF).

### 2.11 Detection of Smad3 Phosphoisoforms by Enzyme‐Linked Immunosorbent Assay (ELISA)

A capture ELISA was developed to detect the amount of cytosolic and nuclear quantities of specific linker and c‐terminal phosphoisoforms of Smad3 in AoSMC infected with *P. gingivalis*. ELISAs were performed with the Invitrogen Antibody pair buffer kit (Catalog # CN B0011) according to the manufacturer's instructions. Mouse anti‐Smad2/3 (Santa Cruz, Biotechnology Inc., Catalog # sc‐398844, RRID: AB_2941302), which recognizes the n‐terminal sequence of Smad2/3, was used for Smad3 capture at a dilution of 1:500 in coating buffer B. The following rabbit antibodies used for detection of linker phosphorylated Smad3 (pSmad) included rabbit anti‐pSMAD3L^(Ser204)^ (1:500 dilution, Catalog # bs‐5235R, RRID: AB_11056215, Bioss Antibodies, biossusa.com) and anti‐pSmad3L^(Ser213)^ (1:5000 dilution, Catalog # bs‐5459R, RRID: AB_11043256, Bioss Antibodies, biossusa.com). Phosphorylation of the c‐terminus of Smad3 (pSmad3C) was detected with anti‐pSmad3C^(Ser423+Ser425)^ (1:500 dilution, Bioss, Catalog # bs‐3425R, RRID: AB_10857310). All rabbit antibodies were diluted in blocking buffer. Goat antirabbit IgG (H + L) Cross‐Adsorbed HRP (1:5000 dilution, Catalog # G‐21234, Thermo Fisher Scientific) in blocking buffer was used as the secondary antibody.

AoSMC was serum‐starved and inoculated with *P. gingivalis* prepared in DMEM/F‐12 with 5% FBS as described for proliferation and migration assays. After 24 h, cytosolic and nuclear extracts of AoSMC were prepared with EpiQuik Nuclear Extraction Kit I (Catalog # OP‐0002, Epigentek, Farmingdale, NY) with Halt protease and phosphatase inhibitors cocktail with EDTA (Catalog # 78440, Thermofisher Scientific) and immediately stored at −80°C. Protein concentrations of each extract were determined by Bradford Protein Assay (Catalog # 23246, Pierce Detergent Compatible Bradford Assay Kit, Thermofisher Scientific). For the ELISA, AoSMC cell extracts were diluted with blocking buffer that contained Halt protease and phosphatase inhibitors cocktail with EDTA (Catalog # 78440, Thermofisher Scientific). Each sample was run in duplicate, and all pSmad3 phosphoisoforms for each sample were included on the same ELISA plate. Samples were incubated at 37°C for 2 h, washed, and rabbit anti‐pSmad three antibodies were added and incubated overnight at 4°C. The next day, goat anti‐HRP was added to each sample, and plates were incubated at room temperature for 30 min and washed three times before the addition of 3,3′,5,5′‐tetramethylbenzidine substrate. Stop solution was added to each well after 40 min, and plates were read with a microplate reader (Bio‐Rad Laboratories, Model 680) at 450 nm. The average absorbance value of each sample was normalized by the protein concentration of the sample. To adjust for interplate/interexperiment variation, data from each experiment were converted to fold change by dividing the absorbance/mg protein of infected samples by the absorbance/mg protein of the corresponding uninfected control.

### 2.12 Detection of Nuclear pSmad3 Phosphoisoforms in AoSMC by Microscopy

AoSMC was seeded onto chamber slides (NUNC Lab‐Tek II Chamber Slide system, Thermo Fisher Scientific, Catalog # 154534), serum‐starved and inoculated with *P. gingivalis* prepared in DMEM/F‐12 with 5% FBS as described for proliferation and migration assays. After 24 h, *P. gingivalis*‐inoculated AoSMC was fixed, permeabilized, and blocked as described for the proliferation assay. After blocking, AoSMC was incubated with rabbit anti‐pSmad3L(Ser204) (1:200 dilution, Catalog # bs‐5235R, RRID: AB_11056215, Bioss) + mouse anti‐pSmad3(Ser425) (1:100 dilution Catalog # M00059S425, RRID: AB_2941303, Bosterbio) or anti‐pSmad3L(Ser213) (1:5000 dilution, Catalog # bs‐5459R, RRID: AB_11043256, Bioss) + mouse anti‐pSmad3(Ser425) (1:100 dilution Catalog # M00059S425, RRID: AB_2941303, Bosterbio). Isotype controls received normal rabbit serum (Catalog # 31883, Thermo Fisher Scientific) and mouse IgG isotype control (Catalog # 31903, Thermo Fisher Scientific). Primary antibodies were incubated overnight at 4°C. Secondary antibodies were goat antirabbit ALEXA 647 (Catalog # A‐21244, Life Technologies, Grand Island, NY) and goat antimouse ALEXA 594 (Catalog # A‐11032, Life Technologies, Grand Island, NY), which were incubated for 30 min at room temperature. Nuclei were counterstained with DAPI (Catalog # P36934, ProLong Gold Antifade Mountant with DAPI, Thermo Fisher Scientific). For each biological replicate, 10 calibrated images that spanned the chamber area were captured with an EVOS Auto FL imaging system. The number of nuclei negative for pSmad3, positive for pSmad3L (Ser204 or Ser213), positive for pSmad3C (Ser425), or dual phosphorylated (pSmad3L/C) were determined, and results were reported as a percentage of the total cell number.

### 2.13 Statistical Analysis

Graphical plotting of raw values was used to visually assess normality because of small sample sizes per group (https://datatab.net/tutorial/test‐of‐normality), verifying normal distribution. Sample sizes are noted in the figure legends. Quantitative data were analyzed by one‐way analysis of variance (ANOVA) followed by a Tukey multiple comparison test if ANOVA indicated a significant difference among group means. Raw data measurements were log transformed before ANOVA testing if the Bartlett test or Brown–Forsythe test showed variances were significantly different among groups. Testing was performed with Prism 10.0.0 software (GraphPad Prism, www.Graphpad.com). For all testing, *p* < 0.05 was considered significantly different.

## 3 Results

### 3.1 Deletion of PorU in *P. gingivalis* Strain A7UF Inactivates T9SS Function But Does Not Disrupt Expression of the Companion Proteins Found in the Attachment Complex PorUVZQ

**Figure 1:**
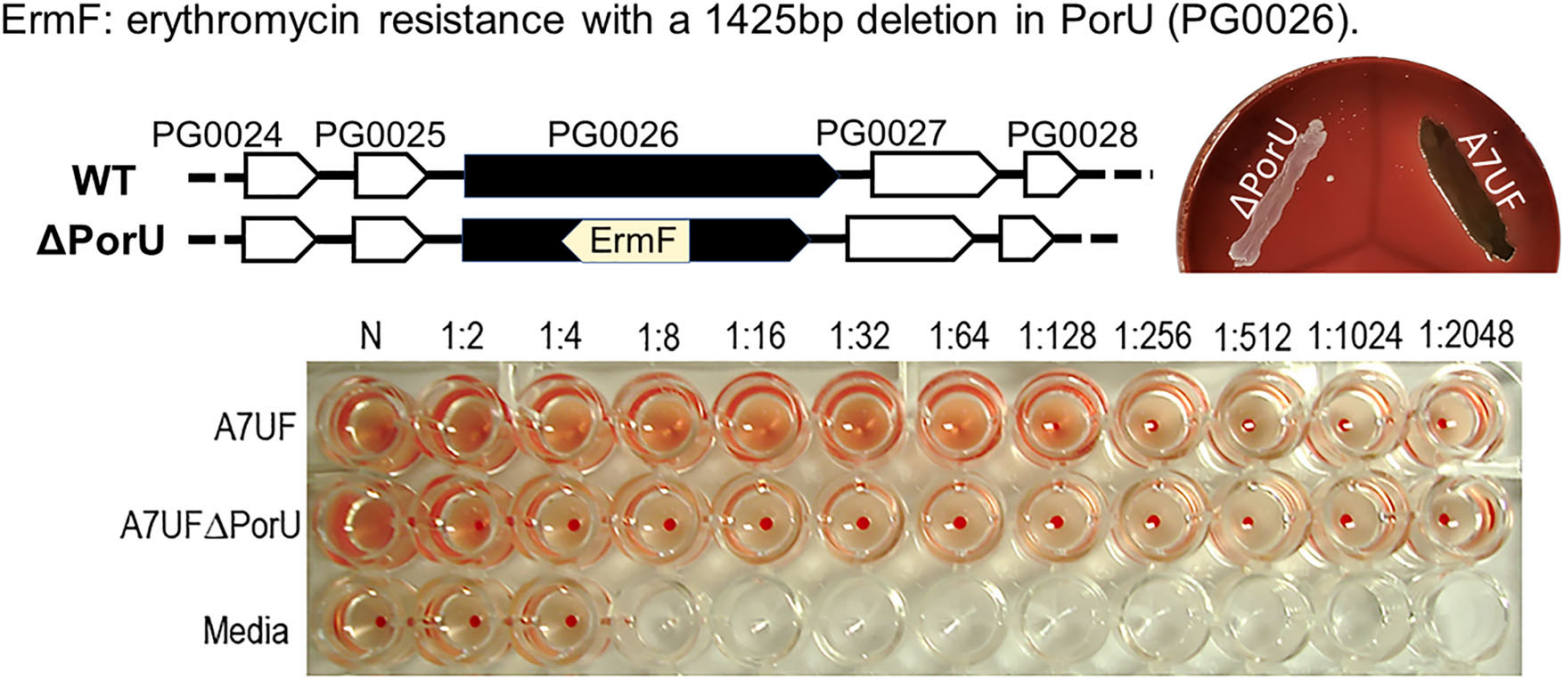
Deletion of the PorU gene in A7UF disrupts T9SS function. The top left diagram shows the insertion and orientation of the erythromycin resistance (ErmF) in the PorU gene that was confirmed by sequencing. Consistent with (Glew et al. 2012) A7UFΔPorU produces unpigmented colonies on supplemented Tryptic Soy blood agar (7 days of growth) and decreased hemolytic activity (bottom picture). T9SS, type 9 secretion system; WT, wild‐type.

As previously described (Glew et al. 2012), disruption of *porU* resulted in loss of pigmentation in A7UFΔPorU colonies grown on sBAP (Figure 1). Hemagglutination/hemolytic activity of A7UFΔPorU was also severely compromised (Figure 1). Because we could not complement PorU in our mutant, likely due to reduced fitness compared with wild‐type (e.g., attenuated for growth, increased sensitivity to freeze storage, and poorly survived the process of electroporation) that prevented introducing DNA constructs into the mutant to restore function, we had our mutant sequenced to validate no unintended mutations. After analyzing the whole genome sequencing data of A7UFΔPorU, we found it was a clean deletion.

To assess for possible polar effects of our deletion mutation, we also reviewed the NanoLC‐MS/MS mass spectroscopy data set from the clarified supernatants. Mass spectroscopy data from the A7UFΔPorU mutant detected protein expression of all the attachment complex proteins PorUVZQ (PG0026, PG0027, PG1604, and PG0602) except PorU. The presence of PorV in the A7UFΔPorU protein fraction indicated there were no polar effects from the deletion of *porU*, since *porV* is located immediately downstream of *porU* on the genome.

### 3.2 T9SS Is Essential for *P. gingivalis* Invasion and Persistence in AoSMC

**Figure 2:**
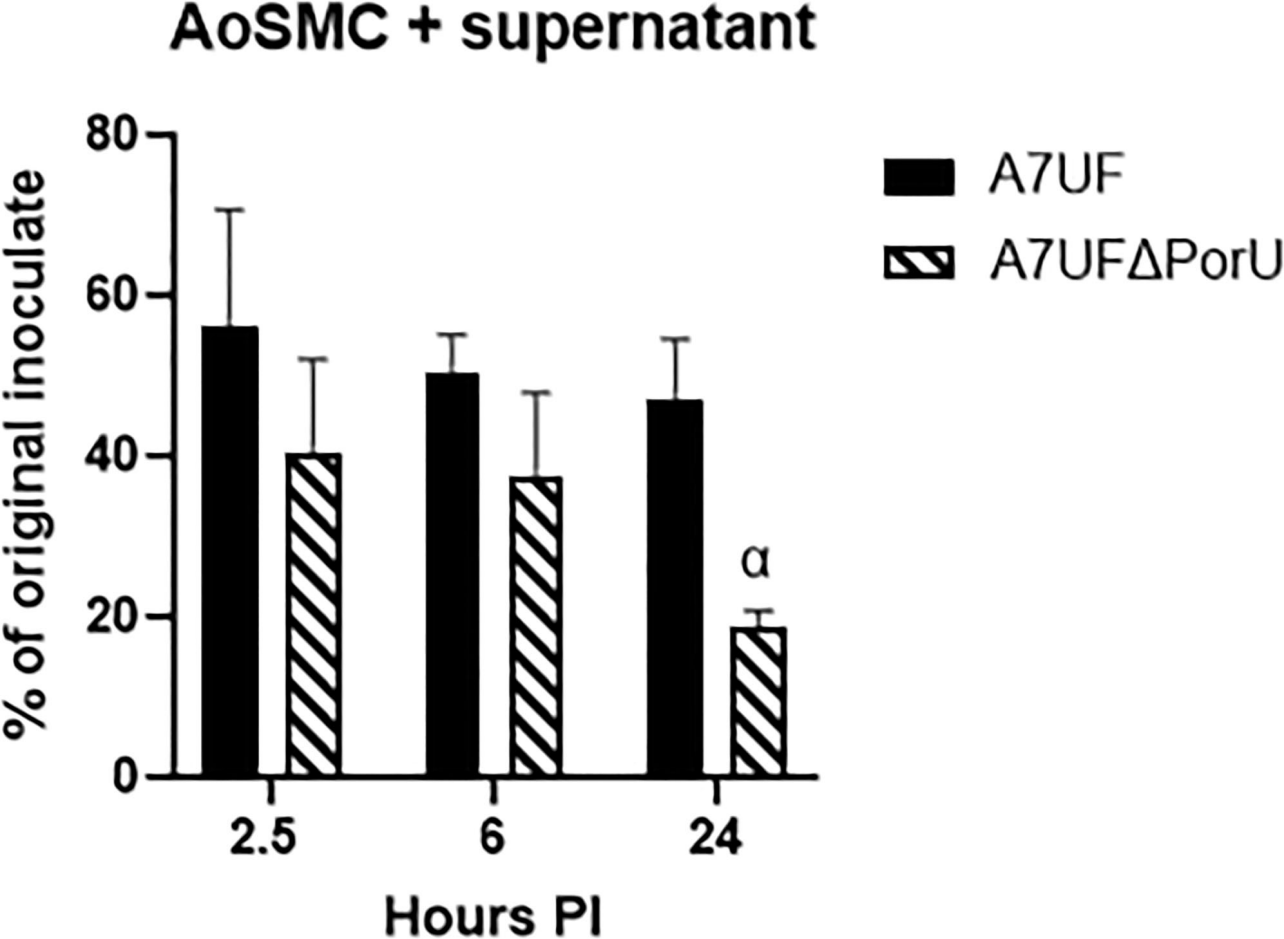
Inactivation of T9SS reduces *P. gingivalis* fitness during in vitro infection of AoSMC. Bacterial survival in AoSMC cocultures harvested at 2.5‐, 6‐, and 24‐h PI (AoSMC + supernatant). Values represent the mean % ± SD of three biological replicates from three separate experiments. α indicates *p* < 0.01 by Student's *t* test at each time point. AoSMC, aortic smooth muscle cell; PI, postinoculation; T9SS, type 9 secretion system.

Since wild‐type *P. gingivalis* is known to invade vascular smooth muscle cells (B. Zhang et al. 2013), we assessed the ability of A7UFΔPorU to colonize, invade, and survive in AoSMC. In our initial infection assays, we enumerated the number of live bacteria in AoSMC combined with their culture supernatant (i.e., included both internalized and extracellular bacteria). Bacterial numbers were expressed as percent of the original colony‐forming unit (CFU) of the corresponding inoculate (Figure 2). There was no difference in the total number of bacteria isolated from AoSMC cultures at 2.5‐ and 6‐h PI, whether extracellular or intracellular. However, by 24‐h PI, the number of A7UFΔPorU retrieved from AoSMC cultures was reduced compared with A7UF (*p* < 0.01).

We next determined the capacity of A7UF and A7UFΔPorU to invade AoSMC by culturing AoSMC supernatants and cell lysates separately after pulse treatment with antibiotics. In these invasion assays, AoSMC culture supernatants from infected wells collected at 2.5 h and centrifuged to remove antibiotics failed to grow bacteria, confirming that antimicrobial treatment was effective in killing extracellular bacteria. The log CFU ± SD of A7UF retrieved from AoSMC lysates collected at 2.5‐h PI was 3.56 ± 0.18 (*n* = 6, two separate experiments). No A7UFΔPorU was retrieved from any AoSMC lysates (*n* = 6) collected at 2.5‐h PI, showing that inactivation of T9SS impaired the ability of *P. gingivalis* to invade and/or survive within AoSMC.

### 3.3 Direct T9SS/AoSMC Interactions Drive *P. gingivalis*‐Induced AoSMC Proliferation

**Figure 3:**
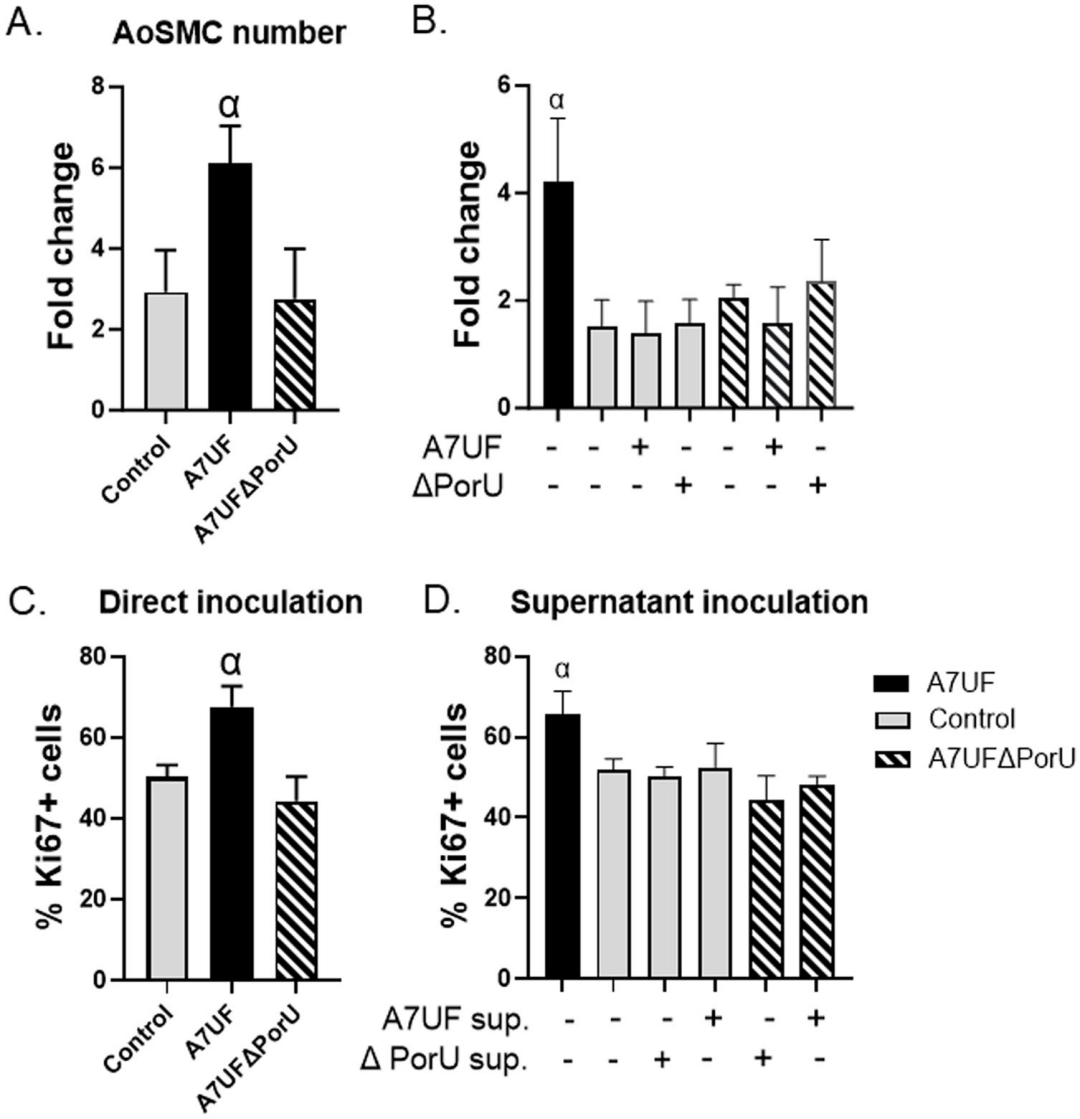
Direct T9SS/AoSMC interactions drive *Porphyromonas gingivalis*‐induced AoSMC proliferation. (A) Number of AoSMCs at 24 h following direct inoculation with sterile media (control, gray bar), or 10 MOI with A7UF (black bar), or A7UFΔPorU (striped bar). Total cell counts were divided by the number of AoSMCs seeded in each well and expressed as the mean ± SD of four biological replicates from two separate experiments. α indicates *p* < 0.05 by one‐way ANOVA and Tukey's multiple comparisons test. (B) Number of AoSMCs at 24 h following indirect inoculation (separated by a 0.4‐µm permeable PES membrane) with sterile media (control, gray), or 10 MOI with A7UF (black) or A7UFΔPorU (striped). Total cell counts were divided by the number of AoSMCs seeded in each well and expressed as the mean ± SD of three biological replicates from two separate experiments. α indicates *p* < 0.05 by one‐way ANOVA and Tukey's multiple comparisons test. (C) The proportion of proliferating AoSMC (Ki67+) at 24‐h postinoculation (PI) with sterile media (gray) or 10 MOI of A7UF (black) or A7UFΔPorU (striped). Values represent the mean ± SD of three biological replicates from two separate experiments. α indicates *p* < 0.05 by one‐way ANOVA and Tukey's multiple comparisons test. (D) The proportion of proliferating AoSMC (Ki67+) at 24‐h PI with sterile media (gray) or 10 MOI of A7UFΔPorU (striped). Inoculated cells were simultaneously treated with clarified supernatant from A7UF or A7UFΔPorU (equivalent to 40 MOI). AoSMC was directly inoculated with 10 MOI of A7UF (black), or sterile media were included as a positive (black) and negative control (gray). Values represent the mean percent ± SD of four biological replicates from two independent experiments. α indicates *p* < 0.05 by one‐way ANOVA and Tukey's multiple comparisons test. Representative images of isotype control and Ki67 staining are available in Supporting Information File 1, Figure S2. ANOVA, analysis of variance; AoSMC, aortic smooth muscle cell; PES, polyethersulfone; T9SS, type 9 secretion system.

We assessed the impact of T9SS function on AoSMC proliferation since wild‐type *P. gingivalis* induces AoSMC proliferation in vitro (B. Zhang et al. 2013). To promote cell quiescence, AoSMC was maintained for 24 h in 1% FBS before inoculation with *P. gingivalis*. Five percent FBS was used as the stimulus for cell division and proliferation, which was assessed by fold increase in cell number and nuclear expression of proliferation marker, Ki67. After 24 h of infection, AoSMC infected with wild‐type A7UF showed a significant increase in cell number (Figure 3A, *p* < 0.02) compared with sham‐inoculated controls and A7UFΔPorU‐infected cells. There was no difference between the uninfected control and the A7UFΔPorU‐infected groups. To determine if *P. gingivalis*‐induced proliferation required a direct interaction between the bacterium and host cells, AoSMC was separated from *P. gingivalis* by a permeable 0.4‐µm membrane (Figure 3B). AoSMC proliferation was detected by positive nuclear staining for Ki67 after cell fixation. Only AoSMCs that were directly inoculated with A7UF showed a significant increase in the proportion of proliferating cells compared with all other groups (*p* < 0.02). To rule out the possibility that *P. gingivalis* OMVs failed to reach AoSMC, uninfected AoSMC and AoSMC infected with A7UFΔPorU were cocultured with clarified supernatant from A7UF or A7UFΔPorU. AoSMC directly inoculated with A7UF was included as a positive control. After 24 h, AoSMCs from each treatment group were counted (Figure 3C). Again, only AoSMC directly inoculated with A7UF (positive control) showed a significant increase in the proportion of proliferating cells (*p* < 0.05). In summary, *P. gingivalis*‐induced proliferation of AoSMC depends on a direct interaction between AoSMC and T9SS components and/or T9SS cargo expressed on the bacterial surface.

### 3.4 *P. gingivalis* T9SS Cargo Are Responsible for Impaired AoSMC Migration

We have previously shown that intrauterine infection with *P. gingivalis* A7UF disrupts the physiologic migration of rat vascular smooth muscle cells from uterine spiral arteries during pregnancy (P. Phillips et al. 2018; Tavarna et al. 2020). To determine if T9SS engages in impaired migration of AoSMC, we used a Transwell system to assess their migration. At the time of inoculation, sham control and infected AoSMC were maintained in 5% FBS for 24 h before transfer to Transwell inserts. Vitronectin, which is abundant in injured vessels as well as the pregnant uterine microenvironment (Ruck et al. 1994; Mangale et al. 2008), was used to stimulate AoSMC migration. To better mimic growth factor conditions of pregnancy, uninfected and infected AoSMC was maintained with 5% FBS in DMEM/F‐12 media for 24 h before transfer to Transwell inserts.

**Figure 4:**
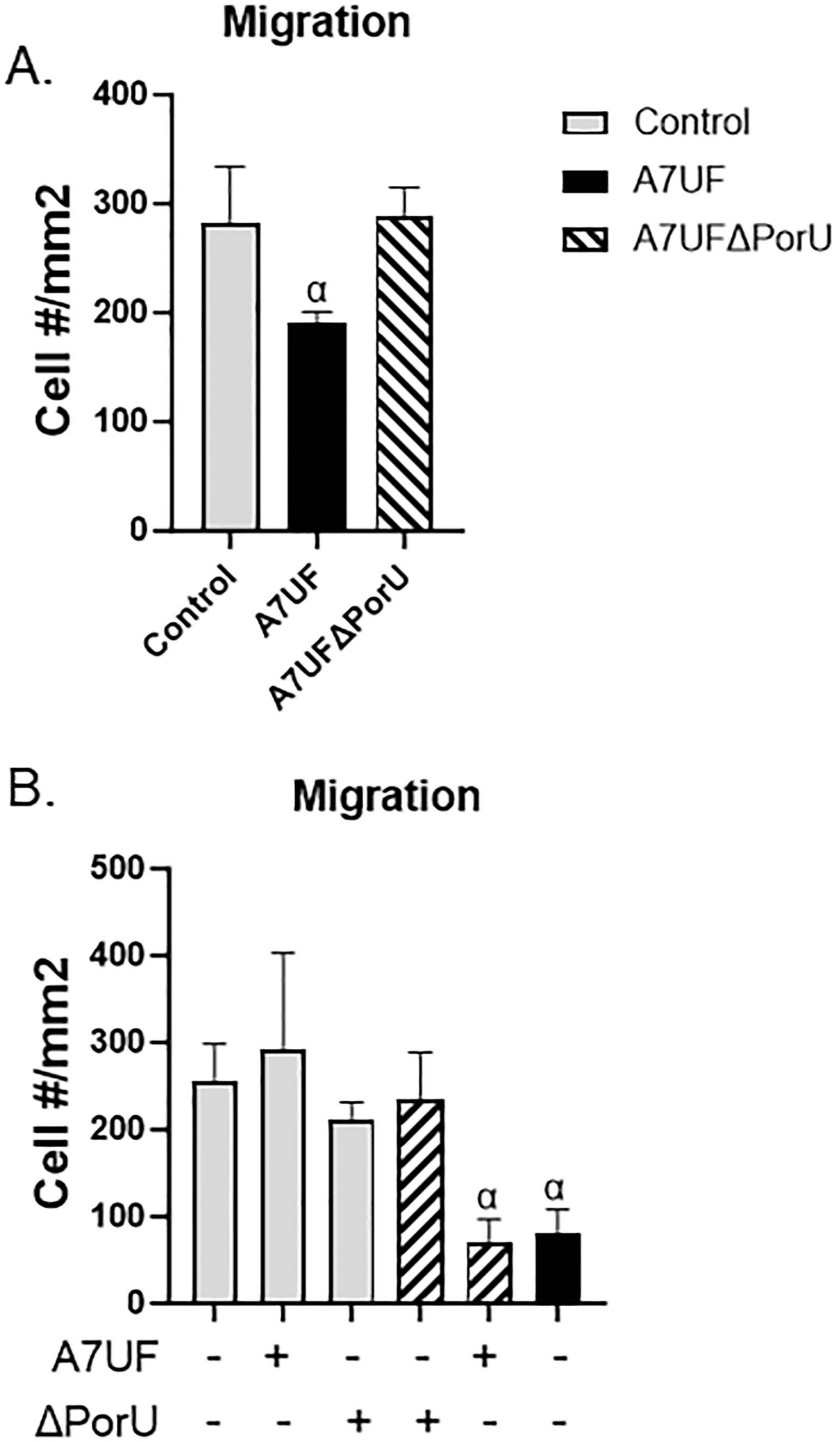
*Porphyromonas gingivalis* T9SS cargo is responsible for impaired AoSMC migration during infection. (A) Number of migrated control and infected AoSMCs after 5 h exposure to vitronectin. Values are expressed as the mean number of cells/membrane area (mm^2^) ± SD of three biological replicates from two separate experiments. (B) Number of migrated control and A7UFΔPorU‐infected AoSMC after 5 h exposure to vitronectin. At the time of inoculation, AoSMC was cocultured with sterile sTSB broth or clarified supernatants from A7UFΔPorU or A7UF. A7UF‐infected AoSMC was included as a positive control. Values are expressed as the mean number of cells/membrane area (mm^2^) ± SD of three biological replicates from two separate experiments. α indicates *p* < 0.05 by one‐way ANOVA and Tukey's multiple comparisons test. ANOVA, analysis of variance; AoSMC, aortic smooth muscle cell; sTSB, supplemented Tryptic Soy Broth; T9SS, type 9 secretion system.

Only AoSMC infected with wild‐type A7UF showed a significant reduction in cell migration (*p* < 0.05) compared with uninfected controls and cells infected with A7UFΔPorU (Figure 4A). To determine if T9SS cargo affects AoSMC migration, clarified supernatant from A7UF or A7UFΔPorU was added to control cultures and A7UFΔPorU cultures at the time of inoculation. AoSMC was directly inoculated with 10 MOI of A7UF and was included as a positive control. After 24 h, cells were evaluated for migration in response to vitronectin (Figure 4B). Neither clarified supernatant from A7UF nor A7UFΔPorU affected AoSMC migration in the uninfected control groups. However, A7UFΔPorU‐infected AoSMC cocultured with clarified supernatant from A7UF showed a significant reduction in migration to vitronectin (*p* < 0.05). Our results suggest an interaction effect between infection with A7UFΔPorU and clarified supernatant from A7UF is necessary for impaired AoSMC migration to vitronectin.

Since an *n* = 1 only reveals a conditionally specific single snapshot of proteomic differences between A7UF and A7UFΔPorU, we restricted our focus to proteins that were uniquely present in the A7UF clarified supernatant. The complete list of proteins identified in the supernatant of A7UF and/or A7UFΔPorU is available in Supporting Information File 2, Table S2. We found three proteins unique to the wild‐type A7UF supernatant. As expected, PorU was only detected in the A7UF sample. Immunoreactive 46 kDa antigen PG99, which is a T9SS cargo protein of unknown function, was present in the A7UF protein fraction and was missing in the A7UFΔPorU sample. This T9SS cargo protein may be trapped in the periplasm of the mutant due to incomplete T9SS cargo processing. Also unique to the A7UF sample was the presence of branched‐chain‐amino‐acid transaminase (ilvE), which is critically involved in both amino acid biosynthesis and degradation in many bacteria and is not a T9SS cargo protein in *P. gingivalis*. The inability to detect ilvE in the A7UFΔPorU sample potentially correlates with the growth restrictions observed in this mutant.

### 3.5 *P. gingivalis* T9SS Perturbs the TGF‐β/Smad Signaling Axis in AoSMC

The TGF‐β/Smad signaling axis is an important regulator of vascular smooth muscle cell phenotype switching (Rezaei et al. 2012) that is also perturbed by wild‐type *P. gingivalis* (B. Zhang et al. 2013). Moreover, the physiologic effect of TGF‐β on vascular smooth muscle cells can be altered through signaling crosstalk, particularly pathways that phosphorylate different sites on TGF‐β effectors, Smad2, and Smad3 (Kamato et al. 2020). Therefore, we utilized a TGF‐β signaling phospho‐array to identify signaling pathways that interact with TGF‐β that are affected by A7UF or A7UFΔPorU (Supporting Information File 3, Table S3). To approximate conditions that matched proliferation and migration assays, sham, and *P. gingivalis*‐inoculated AoSMC was incubated for 24 h before collection and analysis. However, to avoid the confounding effects of growth factors present in FBS that trigger TGF‐β signaling (Lee et al. 2022), AoSMC cultures were maintained in 1% FBS throughout the study. A complete list of all phosphorylated proteins and their intensity ratios is available in Supporting Information File 3, Table S3.

Although we confirmed that live bacteria were present in AoSMC culture supernatants, we were unable to enumerate their numbers. Therefore, we first screened for protein phosphorylation patterns that reflect opposite *P. gingivalis*‐induced changes between A7UF and A7UF∆PorU). Ten AoSMC proteins in infected samples showed opposite phosphorylation patterns that differed by 20% or more compared with the control (Table 1). On the basis of these criteria, inoculation with A7UFΔPorU increased phosphorylation of mTOR^(Ser2481)^, PKCδ^(Thr505)^, and Ras‐GRF1^(Ser916)^ compared with sham and/or A7UF‐inoculated groups. In contrast, inoculation with A7UF∆PorU reduced phosphorylation of Abl1^(Thr754/735)^, c‐Abl^(Tyr412)^, PKCθ^(Ser676)^, Shc^(Tyr349)^, and SP1^(Thr739)^ compared with sham‐inoculated AoSMC. Except for SP1^(Thr739)^, inoculation with A7UF had a nominal effect on these AoSMC proteins. A7UF∆PorU had a nominal effect on Smad1^(Ser465)^, Smad2^(Thr220)^, and Smad3^(Ser204)^, which were markedly increased in AoSMC exposed to A7UF.

**Table 1:** Proteins with opposing phosphorylation states in A7UF and A7UFΔPorU‐infected AoSMC.

Phosphorylated protein	Biological effect of phosphorylation	A7UF/control ratio	A7UFΔPorU/control ratio	A7UFΔPorU/A7UF ratio
mTOR^(Ser2481)^	Activates mTOR kinase activity (Copp et al. 2009)	0.82	1.11	1.35
PKCδ^(Thr505)^	Activates the kinase activity of PKCδ (Jackson and Foster 2004)	0.81	1.50	1.85
RAS‐GRF1^(Ser916)^	Activates Ras signaling (H. Yang et al. 2003)	0.94	1.47	1.47
Abl1^(Thr754/735)^	Favors cytoplasmic localization of Abl1 (Colicelli 2010)	1.04	0.77	0.77
c‐Abl^(Tyr412)^	Activates catalytic domain of c‐Abl (Dorey et al. 2001)	1.11	0.77	0.7
PKCθ^(Ser676)^	Modulates kinase activity (Wang et al. 2012)	1.12	0.72	0.72
Shc^(Tyr349)^	Facilitates activation of MAPK and PI3K/Akt cascades (Wills and Jones 2012)	1.08	0.76	0.76
Smad1^(Ser465)^	Antagonizes canonical TGF‐β signaling (Huang et al. 2018; Wrighton et al. 2009)	1.32	0.97	0.74
Smad2^(Thr220)^	Alters Smad2‐mediated gene transcription (Kamato and Little 2020)	1.53	0.96	0.96
Smad3^(Ser204)^	Enhances binding to CREB binding protein (Millet et al. 2009), altering transcriptional activity of Smad3 in response to TGF‐β (Browne et al. 2013; Huang et al. 2018; Wrighton et al. 2009)	1.36	0.83	0.61
SP1^(Thr739)^	Protects Sp1 from ubiquitin‐dependent degradation, promoting cell proliferation (Tan and Khachigian 2009)	1.25	0.71	0.57

Abbreviations: AoSMC, aortic smooth muscle cell; cAMP, cyclic adenosine monophosphate; CREB, cAMP response element binding; MAPK, mitogen‐activated protein kinase; mTOR, mammalian target of rapamycin; PKC, protein kinase C; TGF‐β, transforming growth factor β.

Eight AoSMC proteins showed increased phosphorylation to both A7UF and A7UFΔPorU (Table 2). However, the degree of phosphorylation was more pronounced in cells inoculated with A7UFΔPorU. Affected protein sites included kinases (Akt2, Erk1^Tyr204^, mTOR^Ser2448^, PAK1^Ser204^, and PAK2^Ser192^), and transcription factors (c‐Myc^Ser62^ and Smad2/3^Thr8^).

**Table 2:** Proteins with increased phosphorylation in A7UFΔPorU‐infected cells compared with the A7UF‐infected group.

Phosphorylated protein	Biological effect of phosphorylation	A7UF/control ratio	A7UFΔPorU/control ratio	A7UFΔPorU/A7UF ratio
AKT2^(Ser474)^	Activates mTOR kinase activity (Copp et al. 2009)	1.02	1.28	1.25
ERK1^(Tyr204)^	Regulation of ERK1 kinase activity (Wortzel and Seger 2011)	1.74	2.14	1.23
mTOR^(Ser2448)^	Activates mTOR kinase activity (Copp et al. 2009)	1.18	1.78	1.51
PAK1^(Ser204)^	Contributes to kinase activation (Hammer and Diakonova 2015)	1.10	1.57	1.43
PAK2^(Ser192)^	Contributes to kinase activation (Kouwenhoven et al. 2013)	1.23	1.49	1.22
Myc^(Ser62)^	Targets Myc for proteosomal degradation (Yada et al. 2004)	1.04	1.43	1.38
Smad2/3^(Thr8)^	Can inhibit Smad3 transcriptional activity when Thr179 and Ser213 are also phosphorylated (Matsuura et al. 2004)	1.58	1.96	1.24

Abbreviations: ERK1, extracellular signal‐regulated kinase 1; mTOR, mammalian target of rapamycin.

AoSMC inoculated with A7UFΔPorU showed a profound reduction of phosphorylation at 8 protein sites, which was ≥ 20% reduction when compared with the A7UF‐inoculated group (Table 3). Affected sites included kinases (Akt1^(Ser124 and Tyr326)^, p38 MAPK^Tyr182^, PAK1/2/3^(Thr423/402/421)^, and the regulatory subunit p85 of PI3‐kinase), the actin binding protein cofilin^Ser3^, and transcription factor Smad1^Ser187^.

**Table 3:** Proteins with decreased phosphorylation in A7UFΔPorU‐infected cells compared with the A7UF‐infected group.

Phosphorylated protein	Biological effect of phosphorylation	A7UF/control ratio	A7UFΔPorU/control ratio	A7UFΔPorU/A7UF ratio
AKT1^(Tyr326)^	Enhances AKT activity (Chan et al. 2014)	0.86	0.58	0.67
AKT1^(Ser124)^	Currently unknown (Guo et al. 2014)	0.65	0.33	0.5
Cofilin^(Ser3)^	Suppresses cofilin function (Canovas and Nebreda 2021)	0.68	0.41	0.61
ERK1^(Thr202)^	Regulation of ERK1 kinase activity (Wortzel and Seger 2011)	1.8	1.3	0.7
P38 MAPK^(Tyr182)^	Activates kinase activity (Canovas and Nebreda 2021)	0.36	0.23	0.63
PAK1/2^(Ser199)^	Modulates PAK interactions with SH3 proteins (Chong et al. 2001)	0.69	0.52	0.74
PAK1/2/3^(Thr423/402/421)^	Activates PAK (King et al. 2000)	0.47	0.34	0.74
PI3 kinase p85 α/γ^(Tyr467/199)^	Binds to activated protein‐Tyr kinases and regulates their kinase activity (Liu et al. 2009)	0.29	0.20	0.7
SEK/MKK4^(Ser80)^	Downregulates activity (Spillman et al. 2007)	0.41	0.19	0.47
Smad1^(Ser187)^	Inhibits nuclear accumulation of Smad1 (Kretzschmar et al. 1997)	0.77	0.61	0.61

Abbreviations: ERK1, extracellular signal‐regulated kinase 1; PAK, p21‐activated kinase; SH3, Src Homology 3.

Seventeen AoSMC proteins showed a similar phosphorylation pattern to inoculation with A7UF or A7UFΔPorU (e.g., ≤ 10% difference compared with control). These phosphorylation sites are listed in Supporting Information File 3, Table S3. Taken together, the different and similar phosphorylation patterns listed in Tables 1, 2, 3 suggest that inactivation of T9SS has a biological effect on the TGF‐β/Smad signaling axis.

### 3.6 *P. gingivalis* T9SS Alters Smad3 Phosphorylation in AoSMC

To correlate changes in Smad3 phosphorylation with *P. gingivalis*‐induced AoSMC proliferation experiments, we evaluated the impact of T9SS on TGF‐β/Smad signaling in the presence of 5% FBS. Not only did this replicate the same conditions used in our proliferation experiments, but it also took into consideration that growth factors present in the higher concentration of FBS could alter the outcome of TGF‐β signaling (Davidson et al. 1993). We focused on Smad3 because its phosphorylation state showed one of the most profound effects on T9SS (Table 1), and linker phosphorylation of Smad3 has been shown to enhance epithelial cell proliferation in response to TGF (Matsuzaki 2011). Smad3 phosphorylation was evaluated at 2.5‐h PI when A7UF but not A7UFΔPorU invaded AoSMC, and at 24‐h PI when AoSMC proliferation was enhanced in A7UF‐inoculated cells. Smad3 phosphorylation in the linker region at Serine^204^ and Serine^213^ as well as c‐terminal phosphorylation at Serine^423/425^ was analyzed. Both nuclear and cytosolic fractions were evaluated by ELISA since nuclear translocation of Smad is consistent with active transcriptional regulation (Matsuzaki 2011).

**Figure 5:**
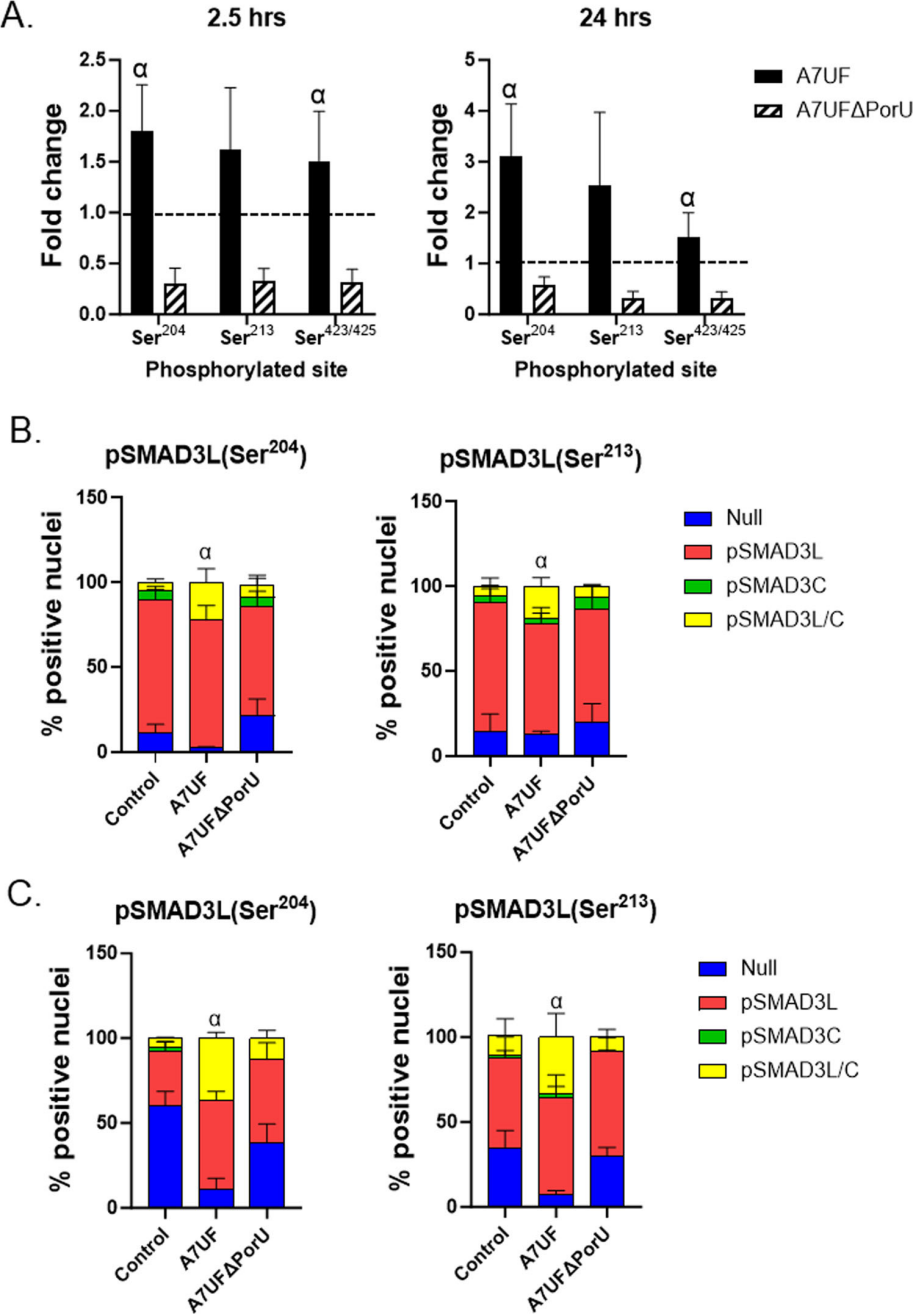
Deletion of PorU attenuates *Porphyromonas gingivalis*‐induced linker and dual phosphorylation of Smad3. (A) Fold change in phosphorylated Smad3 in AoSMC extracts obtained at 2.5‐ and 24‐h postinoculation (PI). Values represent the mean ± SD of three biological replicates from three separate ELISA experiments. Fold change was determined by dividing ABS/mg protein of infected samples by the ABS/mg protein of the corresponding sham‐inoculated control. α indicates values that were significantly different (*p* < 0.01 by Student's *t* test). The proportion of AoSMC nuclei positive for linker (pSmad3L), c‐terminal (pSmad3C), or dual phosphorylated (pSmad3L/C) at 2.5 h (B) and 24‐h PI (C). Null refers to nuclei negative for any Smad3 phosphoisoform. Representative images of pSmad3 phosphorylation are available in Supporting Information File 1, Figure S4. Values represent the mean ± SD of three biological replicates from three separate experiments. α indicates a significant increase in the proportion of pSmad3L/C positive nuclei (*p* < 0.05) determined by ANOVA, followed by Tukey's multiple comparisons test. ABS, absorbance; AoSMC, aortic smooth muscle cell; ELISA, enzyme‐linked immunosorbent assay.

At 2.5‐h PI, A7UF significantly enhanced Smad3 phosphorylation at Ser^204^ and Ser^423/425^ in the nuclear fraction of AoSMC (*p* < 0.05) that persisted in the 24‐h PI group (Figure 5A). The degree of linker phosphorylation at Ser^213^ by the A7UF group tended to be elevated, but it was not significant. In contrast, the nuclear fraction of AoSMC inoculated with A7UFΔPorU had reduced phosphorylation of Smad3 at Ser^204^ and Ser^423/425^ compared with A7UF‐inoculated cells. There was no difference in the amount of Smad3 phosphoisoforms within the cytosolic fraction of AoSMC inoculated with either A7UF or A7UFΔPorU (Supporting Information File 1, Figure S3).

Since dual phosphorylation of Smad3 (i.e., linker + c‐terminus) is implicated in pathologic proliferation in response to TGF‐β (Matsuzaki 2011; Yamaguchi et al. 2013), we performed colocalization analysis of Smad3 phosphoisoforms in sham and *P. gingivalis*‐inoculated AoSMC. Nuclear colocalization of phosphorylated Smad3 at Ser^204^ + Ser^423/426^, and Ser^213^ + Ser^423/426^ was performed on Inoculated AoSMC harvested at 2.5 (Figure 5B) and 24‐h PI (Figure 5C). At 2.5‐h PI, the A7UF group showed a significant increase the proportion of double positive nuclei (i.e., Ser^204^/Ser^423/425^ and Ser^213^/Ser^423/425^) compared with control and A7UFΔPorU groups. By 24‐h PI, the proportion of dual phosphorylated AoSMC in the A7UF group was further increased, showing a sustained activation of Smad3 that was not present in either control or A7UFΔPorU groups. At both time points, AoSMC inoculated with A7UFΔPorU showed a similar Smad3 phosphorylation pattern compared with controls. Taken together, these results show that *P. gingivalis* T9SS and/or T9SS cargo play a role in sustained dysregulation of Smad3 phosphorylation in AoSMC.

## 4 Discussion

To date, defining the function of T9SS in *P. gingivalis* has been largely restricted to identifying the essential components of this system and understanding how this system processes T9SS cargos (Lasica et al. 2017; Mizgalska et al. 2021; Song et al. 2022; Veillard et al. 2019). Despite recent advances, the functional characterization of T9SS in the context of pathogen/host interactions is still in its infancy (Braun et al. 2022). Using AoSMC as our in vitro infection/disease model, we have established that a functioning T9SS in *P. gingivalis* is important for optimal microbial colonization, invasion of host cells, and prolonged microbial survival under cell culture conditions. A caveat with our approach is that we did not independently assess the effect the culture conditions may have had on A7UFΔPorU viability in our infection and invasion assays. It has been reported that inactivation of T9SS impairs *P. gingivalis* resiliency to peroxide‐induced oxidative stress (D. Yang et al. 2021). Environmental oxidative stress may have contributed to the observed differential reduction in survival of extracellular viable mutants at 24‐h PI that was not observed at 2.5‐ and 6‐h PI in our infection assays, considering that these assays were performed in a normoxic incubator. However, the solubility of oxygen in media cultured at 37°C and 5% CO_2_ (v/v) is generally understood to be low, theoretically up to the reported 0.4% O_2_ (v/v) typical for blood plasma (Kane 2020), which would be consumed by the host cells and theoretically further reducing its local relative availability. Further, the mutant tolerated atmospheric oxygen exposure during processing to produce the inoculum without detectable reduction in cell counts, indicating the mutant retained some degree of atmospheric oxidative stress tolerance native to *P. gingivalis*. Certainly, any exogenous oxidative stress generated by the AoSMCs would contribute to its resistance to invasion and reduce the extracellular survival of the mutant by 24 h in the culture wells. However, the inability of the mutant, unlike the wild‐type strain, to invade AoSMCs in our invasion assays at detectable levels, which occurs during the 1‐h PI incubation period before pulse antibiotic treatment to kill extracellular bacteria, was demonstrated, and we hypothesize that increased oxidative stress sensitivity is unlikely to be the main contributing factor. Despite this potential limitation, our studies collectively suggest that T9SS components or T9SS cargos play a role in perturbing TGF‐β/Smad2/3 signaling, which could promote dysregulation of vascular smooth muscle cell plasticity.

Our rationale for using the deletion of *PorU* to disrupt T9SS function in *P. gingivalis* was based on several factors. First, deletion of PorU has been shown to disrupt T9SS function in other strains of *P. gingivalis* as evidenced by accumulation of T9SS cargos in the periplasm and impaired function of select T9SS cargos, such as loss of colony pigmentation and decreased hemagglutinating and gingipains activity (Glew et al. 2012; Mizgalska et al. 2021). Second, PorU transpeptidase and sortase activities require the binding of PorU to the T9SS attachment complex (Mizgalska et al. 2021). Third, PorU has one of the strongest binding affinities to AoSMC membrane proteins, and its binding to AoSMC is associated with altered AoSMC plasticity (P. L. Phillips et al. 2022).

As shown in our NanoLC‐MS/MS mass spectroscopy data, all the attachment complex proteins except PorU were detected in the mutant. In terms of the T9SS machinery membrane assembly, the majority of T9SS protein components, including PorV and PorQ of the attachment complex, does not have a CTD signal and thus does not depend on PorU's CTD peptidase function for membrane localization. Exceptions are PorU and PorZ of the attachment complex and PorA of the translocon complex. However, they are group III cargo proteins that typically retain their CTD signal sequence after transport to the outer membrane and use their CTDs to associate with the integral‐membrane proteins of their respective complexes (Yukitake et al. 2020; Mizgalska et al. 2021). Thus, we believe the wide range of phenotypic disruptions observed in our mutant is a consequence of the loss of PorU at the membrane surface and/or terminating the critical functions of PorU within the attachment complex to appropriately process cargo proteins that require CTD cleavage.

Given that proliferation was measured at 24‐h PI, we cannot exclude the possibility that reduced viability of A7UFΔPorU was the cause of attenuated AoSMC proliferation. However, failure of A7UFΔPorU to invade AoSMC at the 2.5‐h time point that coincided with loss of *P. gingivalis*‐mediated changes in Smad phosphorylation could also be a factor. *P. gingivalis* invasion of colorectal cancer cells activates the Erk pathway, stimulating cell proliferation (Mu et al. 2020). This effect was attenuated when colorectal cancer cells were inoculated with a gingipains (T9SS cargo)‐deficient isogenic mutant. In addition to alterations in Smad3 phosphorylation, the TGF phosphor array showed decreased dual phosphorylation of Erk1/2 in AoSMC inoculated with the PorU mutant. Dual phosphorylation of Erk1/2 is necessary for activation of kinase activity (Wortzel and Seger 2011).

We showed that AoSMC required direct interaction with A7UF to induce proliferation and that the PorU mutant was attenuated for this response. It should be noted that purified gingipains extracts have been shown to induce proliferation in rat AoSMC (Cao et al. 2015). Although clarified supernatant from A7UF had protease activity, it was unable to induce AoSMC proliferation when added directly or when A7UF cells were cocultured with AoSMCs separated by a semipermeable membrane. This discrepancy may be due to the methodology where they used concentrated purified Rgp and Kgp extracts ranging from 3 to 100 μg/mL, whereas the Rgp detected in our clarified A7UF supernatant was neither purified nor concentrated (Supporting Information File 2, Figure S5 and Table S2).

The clarified supernatant from A7UF was able to impair the migration of AoSMC infected with the PorU mutant. This observation provided a mechanistic insight into how *P. gingivalis* interferes with the physiologic remodeling of uterine spiral arteries during pregnancy (P. Phillips et al. 2018; Tavarna et al. 2020). In our rat model of *P. gingivalis*‐induced impaired spiral artery remodeling, aggregates of A7UF are consistently found in the decidual/mesometrial stroma, away from poorly remodeled arteries. Whereas detection of A7UF either within or in direct apposition with the spiral arterial wall is a rare finding. It has been noted that T9SS cargos, which are enriched in *P. gingivalis* OMVs (Veith et al. 2014), diffuse across the host tissues locally as well as travel through distant body sites via the circulation (Okamura et al. 2021). Moreover, *P. gingivalis* OMVs perturb cell signaling events in host cells, such as affecting epithelial cell migration and promoting calcification of murine AoSMC (Furuta, Takeuchi, et al. 2009; W. W. Yang et al. 2016).

We restricted our interpretation of the supernatant proteome to proteins that were unique to the A7UF sample, since we only examined one sample per group. Two of the proteins uniquely identified in the A7UF sample are T9SS cargo, PorU, and Immunoreactive 46 kDa antigen PG99. In contrast, other T9SS cargos including Rgp gingipains were detected in the proteome of both A7UF and A7UFΔPorU (Supporting Information File 2, Table S2), but their spectral counts in A7UF were less than or equivalent to A7UFΔPorU. This is not surprising since PorU mutants have been reported to leak unprocessed gingipains into the culture supernatant (Mizgalska et al. 2021). Even though Rgp spectral counts were higher in the A7UFΔPorU sample (158 vs. 87 spectral counts), A7UFΔPorU has diminished proteolytic activity. Therefore, Rgp should still be considered as a candidate for impaired migration based on its effects on epithelial cells (Furuta, Takeuchi, et al. 2009).

Our observation that *P. gingivalis* interfered with AoSMC migration to vitronectin in the presence of FBS appears to contradict our previous results in which wild‐type A7UF enhanced AoSMC migration to vitronectin under serum depletion conditions (P. L. Phillips et al. 2022). Since FBS contains a variety of growth factors, cytokines, hormones, and glycoproteins (Lee et al. 2022), they may be interfering with vitronectin binding to its receptor(s) or vitronectin/integrin signaling by tyrosine kinase crosstalk (Soung et al. 2010).

We also found that a functioning *P. gingivalis* T9SS perturbed TGF‐β/Smad3 signaling in infected AoSMC. Specifically, dually phosphorylated Smad3 was increased in the nucleus of AoSMC infected with wild‐type A7UF. Although our studies do not establish a mechanistic link between perturbed TGF‐β/Smad3 and enhanced AoSMC proliferation, it does provide a rationale for future studies exploring the impact of *P. gingivalis*‐mediated Smad3 phosphorylation on AoSMC function. Overexpression of Smad3 in a rat model of injury‐induced aortic intimal hyperplasia promotes pathogenic vascular smooth muscle cell proliferation (Tsai et al. 2009). A separate study demonstrated that overexpression of Smad3 in vitro and in vivo enhances TGF‐β‐mediated rat vascular smooth muscle cell proliferation through activation of Smad3/ERK MAPK signaling (Suwanabol et al. 2012). Although these studies (Suwanabol et al. 2012; Tsai et al. 2009) did not evaluate Smad3 phosphorylation patterns, it should be noted that linker and dual phosphorylation of Smad3 have mitogenic effects on various epithelial cells from breast and colorectal cancers (Matsuzaki 2011; Tarasewicz and Jeruss 2012). Taken together, future experiments to study the effects of *P. gingivalis*‐induced Smad3 phosphoisoforms on AoSMC function should include overexpression of Smad3 linker region mutants that are resistant to phosphorylation as well as knockdown expression of Smad3.

In this study, we did not pursue further analysis of other AoSMC proteins detected in the TGF Phospho Array that were affected by the presence of functional T9SS, but they deserve mention because they phosphorylate various serine residues in the linker region of Smad3. We found ERK1/2 activation (i.e., dual phosphorylation at Thr^202^ and Tyr^204^) was enhanced in AoSMC inoculated with wild‐type *P. gingivalis* and not the T9SS‐deficient mutant. When fully activated, ERK1/2 phosphorylates Thr^179^, Ser^204^, and Ser^208^ within the linker region of Smad3 (Matsuzaki 2011). While other kinases can also phosphorylate the same or different residues within the linker region of Smad3, such as JNK and p38, these kinases showed a similar or reduced activation, respectively, in AoSMC inoculated with the wild‐type or T9SS‐deficient mutant. A distinguishing feature of ERK1/2 is that it can be activated by gingipains and a variety of membrane receptors (Tyr kinases, G protein–coupled receptors, and ion channels), whereas p38 and JNK are activated by cellular stress, cytokines, and/or cell injury (Wortzel and Seger 2011). Further studies evaluating the role of *P. gingivalis*‐induced ERK1/2 activation in Smad3 linker phosphorylation and AoSMC dysfunction would also be of interest.

We have demonstrated a novel effect of *P. gingivalis* T9SS function on TGF‐β/Smad2/3 signaling that was associated with altered AoSMC plasticity. The TGF‐β/Smad2/3 signaling cascade is central to the regulation of inflammation, immunity, embryogenesis, placentation, and tissue remodeling (Sanjabi et al. 2017; Derynck and Budi 2019). Further, dysregulation of the TGF‐β/Smad2/3 signaling axis underlies a variety of degenerative, inflammatory, and neoplastic diseases (Low et al. 2019; Matsuzaki 2011; Tarasewicz and Jeruss 2012; Yamaguchi et al. 2013). Given the ubiquitous nature of TGF‐β/Smad2/3 signaling in disease, our findings have implications for the contribution of the *P. gingivalis* T9SS to diseases involving inflammation and/or abnormal tissue remodeling, such as periodontal, cardiovascular, neoplastic, and placentation disorders (Z. Zhang, Liu, et al. 2021; Tavarna et al. 2022).

## Author Contributions

**Priscilla L. Phillips:** conceptualization, methodology, validation, visualization, software, formal analysis, data curation, investigation, writing – original draft, writing – review and editing, funding acquisition, resources, supervision. **Leticia Reyes:** conceptualization, investigation, funding acquisition, writing – original draft, methodology, validation, visualization, writing – review and editing, formal analysis, project administration, resources, data curation, supervision.

## Conflicts of Interest

The authors declare no conflicts of interest.

## Supporting information

Supplement File 1‐1 (1).

Supplement File 2.

Supplement File 3.

## Data Availability

The data that support the findings of this study are openly available in Zenodo at https://doi.org/10.5281/zenodo.14805777.
