# Supplementary material for: Porphyromonas gingivalis Type 9 Secretion System Promotes Dysregulation of Vascular Smooth Muscle Cell Plasticity With Perturbed TGF‐β/Smad Signaling
Source: Microbiologyopen. 2025 Oct 22;14(5):e70044. doi: 10.1002/mbo3.70044 (PMC12541887; doi:10.1002/mbo3.70044)
Supplement: Supplementary file 1 — Supplement File 1‐1 (1). [file MBO3-14-e70044-s001.docx]

**Supplement File 1, *P. gingivalis* type 9 secretion system promotes dysregulation of vascular smooth muscle cell plasticity with perturbed TGF-β/SMAD signaling.**

**Priscilla L. Phillips^1*^ and Leticia Reyes^2^**

**Table S1. Primer sequences used for mutant construction.**

| **Primer ID** | **Primer Sequence (5’** → **3’)** | **Template** | **PCR**  **product** |
| --- | --- | --- | --- |
| PG0026UF | CCGGTACTCCGTCAGGGCAA | A7UF  genomic DNA | 908 bp |
| PG0026UR | TGATTGGGTACCGCTCCGAGG |  |  |
| PG0026DF | TGCCGGTATTGCCGAGGTGA | A7UF  genomic DNA | 830 bp |
| PG0026DR | CCATGGAGGCCGTCTGTCCT |  |  |
| PG0026ErmFcomp_F | CCTCGGAGCGGTACCCAATCAATTTGCCAGCCGTTATGCGG | pUF4000 | 1215 bp |
| PG0026ErmFcomp_R | TCACCTCGGCAATACCGGCAGGTACCCCCGATAGCTTCCGC |  |  |

**
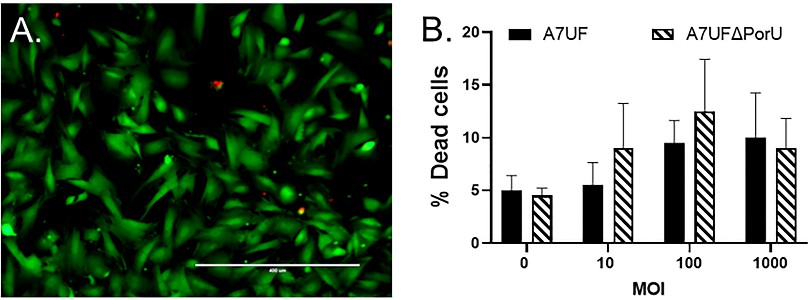
**

**Figure S1.** A) Representative image of live (green) and dead (red) AoSMC**.** B) Mean percent ± SD of dead AoSMC (n = 2) at 24 hours post-inoculation with media, A7UF, or A7UFΔPorU.


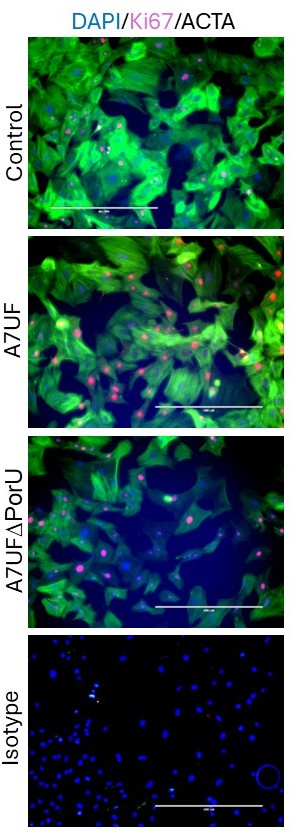


**Figure S2.** Representative images of isotype (normal rabbit serum and mouse IgG isotype control) and Ki67 (red)/ACTA (green) staining that was used to measure AoSMC proliferation. Scale bar = 400 μm.


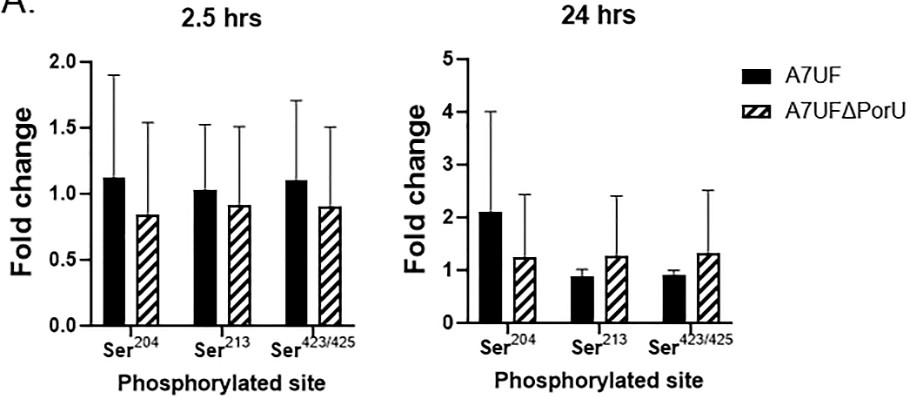

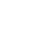


**Figure S3.** Fold change in phosphorylated SMAD3 in AoSMC cytosolic extracts obtained at 2.5- and 24- hours post-inoculation. Values represent the mean ± SD of 3 biological replicates from 3 separate experiments. Fold change was determined by dividing ABS/mg protein of infected samples by the ABS/mg protein of the corresponding sham inoculated control.


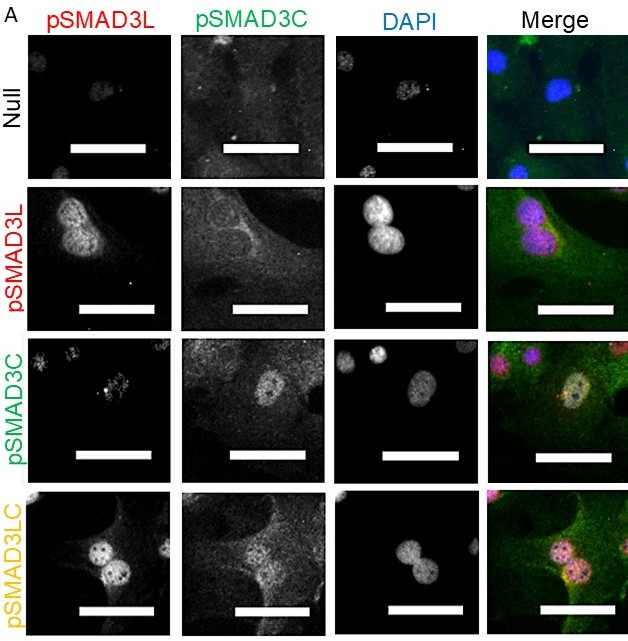


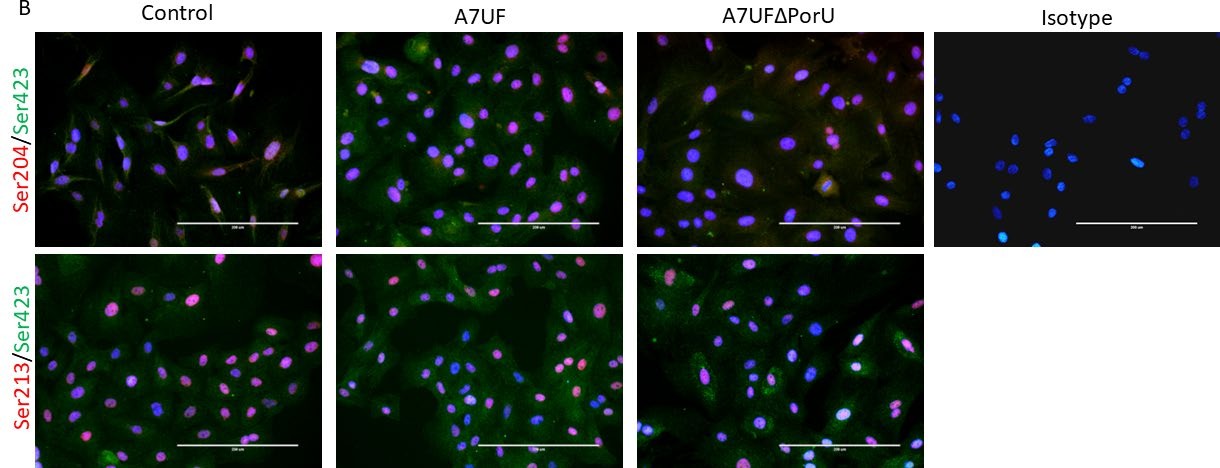


**Figure S4** A) Representative images of criteria used to call a cell Null (only DAPI +), pSMAD3L (at Ser204 or Ser213 only), pSMAD3C (at Ser423/425 only), or pSMAD3LC (both linker and c-terminus +) staining. Black and white images reflect each channel that makes up the merged image. Brightness of the composite image was enhanced with Microsoft Photos software to improve visualization of printed images. Scale bar = 50 µm. B) Raw images of Ser204/ Ser423/425, Ser213/ Ser423/425, and isotype control. Scale bar = 200 μm.
